# Role of extracellular matrix components and structure in new renal models *in vitro*

**DOI:** 10.3389/fphys.2022.1048738

**Published:** 2022-12-07

**Authors:** Alodia Lacueva-Aparicio, Rafael Soares Lindoso, Silvia M. Mihăilă, Ignacio Giménez

**Affiliations:** ^1^ Renal and Cardiovascular Physiopathology (FISIOPREN), Aragon’s Health Sciences Institute, Zaragoza, Spain; ^2^ Tissue Microenvironment Lab (TME Lab), I3A, University of Zaragoza, Zaragoza, Spain; ^3^ Carlos Chagas Institute of Biophysics, Federal University of Rio de Janeiro, Rio de Janeiro, Brazil; ^4^ Division of Pharmacology, Utrecht Institute for Pharmaceutical Sciences, Utrecht University, Utrecht, Netherlands; ^5^ Institute for Health Research Aragon (IIS Aragon), Zaragoza, Spain; ^6^ School of Medicine, University of Zaragoza, Zaragoza, Spain

**Keywords:** extracellular matrix, matrigel, kidney, bioprinting, scaffolds, microfluidics, organoids

## Abstract

The extracellular matrix (ECM), a complex set of fibrillar proteins and proteoglycans, supports the renal parenchyma and provides biomechanical and biochemical cues critical for spatial-temporal patterning of cell development and acquisition of specialized functions. As *in vitro* models progress towards biomimicry, more attention is paid to reproducing ECM-mediated stimuli. ECM’s role in *in vitro* models of renal function and disease used to investigate kidney injury and regeneration is discussed. Availability, affordability, and lot-to-lot consistency are the main factors determining the selection of materials to recreate ECM *in vitro.* While simpler components can be synthesized *in vitro*, others must be isolated from animal or human tissues, either as single isolated components or as complex mixtures, such as Matrigel or decellularized formulations. Synthetic polymeric materials with dynamic and instructive capacities are also being explored for cell mechanical support to overcome the issues with natural products. ECM components can be used as simple 2D coatings or complex 3D scaffolds combining natural and synthetic materials. The goal is to recreate the biochemical signals provided by glycosaminoglycans and other signaling molecules, together with the stiffness, elasticity, segmentation, and dimensionality of the original kidney tissue, to support the specialized functions of glomerular, tubular, and vascular compartments. ECM mimicking also plays a central role in recent developments aiming to reproduce renal tissue *in vitro* or even in therapeutical strategies to regenerate renal function. Bioprinting of renal tubules, recellularization of kidney ECM scaffolds, and development of kidney organoids are examples. Future solutions will probably combine these technologies.

## Introduction

### Extracellular matrix components provide critical cues for renal cell and tissue functions

The extracellular matrix (ECM) is a biological scaffold holding all cellular tissue components together (Supplementary Figure 1). The main components of kidney ECM are collagen I, proteoglycans, and glycosaminoglycans. The basal membrane (BM) surrounding the renal tubules contains collagen IV, laminins, and fibronectin (Theocharis et al., 2016). ECM composition and dimensionality establish biomechanical and biochemical signals essential for kidney’s development (Clause and Barker, 2013; Loganathan et al., 2020), tissue growth, differentiation (Muncie and Weaver, 2018), and function (Frantz et al., 2010; Manninen, 2015; Loganathan et al., 2020).

The stiffness of the ECM influences organ or tissue differentiation and morphogenesis. ECM stiffness is determined by the material’s elasticity, as measured by the Young’s elastic modulus. Conventional plastic cell culture containers (10^9^ Pa) are stiffer than bone (15–20 10^6^ Pa) and the kidney (5–10 10^3^ Pa). The substrate mechanical properties affect cell adhesion, migration, proliferation, and differentiation (Chen et al., 2014; Melica et al., 2019). Topography and dimensionality, which identify ECM forms, features, and distribution, are linked to cell polarization, actin bundle alignment, cell adhesion, orientation, migration, and morphology (Nur-E-Kamal et al., 2006; Kim et al., 2014; Sciancalepore et al., 2016; Hulshof et al., 2018; Bosch-Fortea et al., 2019) and renal progenitors’ fate (Nur-E-Kamal et al., 2006; Kim et al., 2014; Bosch-Fortea et al., 2019; Walma and Yamada, 2020). Microfabrication methods can reproduce ECM mechanical and physical properties and architectural features, but resolution and complexity are still rudimentary (Le Digabel et al., 2010). Engineered ECM can be tailored to meet cell or tissue-specific needs (Beamish et al., 2017).

ECM components, particularly glycosaminoglycans (GAGs), provide biochemical signals that regulate cell functions and the organization of the ECM itself (Weber et al., 2017) (Table 1). Hyaluronan, the most abundant GAG (Iozzo and Schaefer, 2015; Theocharis et al., 2016), heparan sulphate or chondroitin sulphate (Lelongt and Ronco, 2003) are involved in tissue development, by displaying growth factors spatial-temporal distribution during epithelial branching (Nigam and Bush, 2014). ECM-renal cells interact through ECM-binding transmembrane receptors such as integrins (Chen et al., 2004; Clause and Barker, 2013; Handorf et al., 2015; Bülow and Boor, 2019) or polycystins (Nickel et al., 2002) that translate ECM biomechanical features into intracellular signals (Hagelaars et al., 2022). In diabetic nephropathy (Kolset et al., 2012) or autosomal dominant polycystic kidney disease (ADPKD) (Zhang et al., 2020), ECM disruptions result in alterations in kidney function. Changes in ECM synthesis and turnover of laminin, heparan sulphate, and chondroitin sulphate proteoglycans contribute to disease pathogenesis (Zhang et al., 2020). Unresolved renal parenchyma damage causes scarring by abnormal deposition of ECM. Chronic damage or excessive scarring leads to fibrosis, a hallmark of chronic kidney disease (Clause and Barker, 2013; Bülow and Boor, 2019). Any *in vitro* model aiming to accurately represent kidney function, damage, and regeneration should incorporate the ECM compartment. We review ECM’s role in *in vitro* kidney models. Established models are briefly discussed to better understand the advantages of new methodological developments.

**TABLE 1:** Role of ECM in *in vitro* models of renal function and disease.

2D models					
Culture architecture	ECM surrogate	Cells	Biological structure	Application	References
Coated PS plates	Fibronectin, laminin, collagen type IV and Matrigel	hESCs	Differentiated PT-like cells monolayer	Induced Differentiation to PT	Narayanan et al. (2013)
Coated glass plates and electrospun PCL-BU membranes	PCL-BU vs Collagens, laminin, MG, L-Dopa	HK-2, RPTEC	Differentiated PT-like cells monolayer	Synthetic membrane for BAK	van Gaal et al. (2021)
Coated PES/PVP/PSF-FC membranes	Collagen I, collagen IV, Laminin, L-DOPA	HPTCs, HK-2	Differentiated PT-like cells monolayer	Synthetic membrane for BAK	Ni et al. (2011)
Coated PS- and PES-microstructured substrates	L-DOPA, collagen IV	ciPTEC	Monolayer of differentiated PT-like cells	Synthetic membrane for BAK	Hulshof et al. (2018)
Coated microPES hollow fiber membrane	L-DOPA, collagen IV	ciPTEC	Monolayer of differentiated PT-like cells	Synthetic membrane for BAK	Jansen et al. (2015)
Coated PE and PES-50 transwell membrane	L-DOPA, collagen IV	ciPTEC	Monolayer of differentiated PT-like cells	Synthetic membrane for BAK	Schophuizen et al. (2015)
Hydrogel bioprinted onto polyester Transwell membrane	Organovo’s NovoGel Bio-Ink	RPTEC Renal fibroblasts and HUVEC	RPTEC monolayer on top of hydrogel with HUVEC and fibroblasts	Differentiation CTX Fibrosis	King et al. (2017)
Coated Polycarbonate porous membrane within a microchip	Matrigel	RPTECs	Monolayer of differentiated PT-like cells	Synthetic membrane for BAK	Gao et al. (2011)

**2.5D Models**					
**Culture architecture**	**ECM surrogate**	**Cells**	**Biological structure**	**Application**	References
Coated Micropatterned Silicon-PDMS surfaces	Fibronectin, laminin, matrigel. Matrigel in medium	MDCK, RPTEC, LLC-PK1	Cysts and tubules (PT)	Morphogenesis Nephrotoxicity	Bosch-Fortea et al. (2019)
Hydrogels Coated PS plates	Matrigel	HPTCs	Tubules (PT)	Morphogenesis	Zhang et al. (2011)

**3D Models**					
**Culture architecture**	**ECM surrogate**	**Cells**	**Biological structure**	**Application**	References
Hydrogel	20% Growth factor-depleted Matrigel 80%Collagen I	Mouse embryonic UB and BSN primary cells mIMCD3	Cysts and tubules (UB)	Tubulogenesis Development	Sakurai et al. (1997)
Hydrogel	Matrigel	Primary baby mouse kidney epithelial cells	Tubules	Tubulogenesis	Taub et al. (1990)
Hydrogel	Rat tail collagen type I	Primary murine renal cells	Tubule- and glomerulus-like structures	Morphogenesis	Joraku et al. (2009)
0.4 µm Polyester Transwell membranes	Matrigel and collagen I (1:1)	RPTEC	Tubules (PT)	Tubulogenesis	Miya et al. (2011)
0.4 µm polycarbonate Transwell membranes	Matrigel and rat tail collagen I (1:1)	NKi-2	Tubules	Morphogenesis Nephrotoxicity	DesRochers et al. (2013)
Hydrogel	Collagen	MDCK co-cultured with Swiss 3T3	Tubules (Distal nephron)	Morphogenesis	Montesano et al. (1991a)
Hydrogel	Collagen	MDCK and co-cultured with MRC-5	Tubules (Distal nephron)	Morphogenesis	Montesano et al. (1991b)
Hydrogel	Collagen	HK-2	Tubules (PT)	Morphogenesis	Kher et al. (2011)
Hydrogel	Matrigel	Mouse renal tubule fragments	Cysts and tubules (Collecting Duct)	Genetic disease (ADPKD)	Dixon et al. (2020)
Hydrogel	Growth factor reduced, phenol red-free Matrigel	RPTEC/TERT1	Tubules (PT)	Nephrotoxicity	Secker et al. (2018)
Casting molds in 12-well plate	Collagen-Matrigel	Neonatal rat renal cells	Tubule- and glomerulus-like structures	Morphogenesis	Lü et al. (2012)
Round bottom microwell plate	GFR-Matrigel	MDCK	Tubules (distal nephron)	Morphogenesis	Hirashima et al. (2017)
Hydrogel	Collagen I, GRF-Matrigel	RPTEC, renal fibroblasts and HUVEC	Tubules (PT) and endothelial unit	Tubulo-vascular interactions	Wang et al. (2020)
Printed silicon gasket	Gelatin, Fibrinogen	PTECT-TERT1, GMECs	Tubules (PT) and endothelial unit	Epithelial transport Tubulo-vascular interactions	Lin et al. (2019)
Hydrogel	Collagen I	HKC-8 and WS-1	HKC-8 monolayer on top of WS-1 embeded hydrogel	Fibrosis Nephrotoxicity	Moll et al. (2013)
Polystyrene multiwell plate	Covalent polymer networks of heparin and/or starPEG	HK-2	Tubules (PT)	Tubulogenesis	Weber et al. (2017)
PEGDA Hydrogel	HA	Mouse proximal tubule cells	Tubules (PT)	Nephrotoxicity	Astashkina et al. (2012); Astashkina et al. (2014)
Hydrogel	HA Matrigel	Embryonic rat UB	Tubules (UB)	Morphogenesis	Rosines et al. (2007)
PEG hydrogel	PEG functionalized with RGD peptide, laminin-1	MDCK	Cysts	Epithelial morphogenesis	Chung et al. (2008)
Scaffold	Silk	hiPSCs	Organoids	Development Differentiation	Gupta et al. (2019)
Scaffold	Thiol-ene crosslinked alginate	hiPSCs	Organoids	Development Differentiation	Geuens et al. (2021); Ruiter et al. (2022)
Scaffold	PLA Matrigel-Geltrex	HRECs	Monolayer-	ECM biomechanical properties	Love et al. (2019)
Hydrogel	PEG-4-MAL	MDCK	Cysts	ECM biomechanical properties	Enemchukwu et al. (2016)
PCLdi (u-UPy) electro-spun HFM	Collagen I, IV, fibronectin, laminin	hRPTECs	Monolayer	Bioactive membranes for BAKs	Dankers et al. (2011)
Electrospun transwell membrane	1:1 dKECM-PLC	hRPCs HUVEC	Monolayer	Differentiation Tubule-Vascular unit Nephrotoxicity	Sobreiro-Almeida et al. (2019); Sobreiro-Almeida et al. (2020)
Melt-electrowritten tubular scaffold	PCL	ciPTEC HUVEC	Monolayer Self-produced ECM	Tubule-Vascular unit Bioactive membranes for BAKs	van Genderen et al. (2021)
Silk-based porous scaffold	Matrigel and Collagen-Matrigel	MEK	Tubules and cysts	Genetic disease (ADPKD)	Subramanian et al. (2010)
Silk-based porous scaffold	Collagen type I and Matrigel (1:1)	mIMCD	Cysts	Genetic disease (ADPKD)	Subramanian et al. (2012)
Hollow tubes insidehydrogel	Collagen I	MDCK Primary PCT from transgenic mice	Tubules	Genetic disease (ADPKD)	Myram et al. (2021)
Extruded topographic hollow fiber (h- FIBER)	RGD-conjugated alginate	Podocytes and endothelial cells	Tubules Glomerulus-like structure	Glomerular filtration studies	Xie et al. (2020)
EDC hollow fibers	Collagen IV	HK-2	Tubules (PT)	Bioengineering renal tubules	Shen et al. (2015)
MicroPES HFM	Collagen IV and L-DOPA	ciPTEC	Tubules (PT)	Bioactive membranes for BAKs	Chevtchik et al. (2016)
PCL tubular nanofiber scaffold	Collagen IV and L-DOPA	ciPTEC-OAT1	Tubules (PT)	Bioactive membranes for BAKs Nephrotoxicity	Jansen et al. (2019)

**Bioprinted Scaffolds**					
**Culture architecture**	**ECM surrogate**	**Cells**	**Biological structure**	**Application**	References
Bioprinted renal constructs	dKECMMA	Human primary kidney cells	Tubular Glomerular-like structures	Tissue bioengineering	Ali et al. (2019)
Bioprinted renal construct	dKECM Gelatin	hRPCs HUVEC, podocytes	3D glomerular model	Regenerative medicine	Sobreiro-Almeida et al. (2021)
Bioprinted hollow tubules	dECM and alginate	RPTEC, HUVEC, hBMMSCs	Perfused Tubules and capillaries	Regenerative medicine	Singh et al. (2020)
Bioprinted hollow tubules	Gelatin-fibrin hydrogel	RPTEC/TERT1, GMECs	Perfused Tubules and capillaries	Tubule-Vascular unit Nephrotoxicity	Homan et al. (2016); Lin et al. (2019); Aceves et al. (2022)
Hydrogel-sandwiched, bioprinted tubular structure	Collagen I, Matrigel, Fibrin	RPTEC/TERT1, iRECs	Perfused Tubules	Bioengineering renal tubules	Tröndle et al. (2021)

Natural polymers: HA: hyaluronic acid, FMB: fibrin microbreads, dKECMMA: photo-crosslinable kidney ECM-derived bioink.

Synthetic polymers: EDC: 1-ethyl-3-(3- (dimethylamino)propyl) carbodiimide hydrochloride, PA: polyacrylamide.

Cell lines: HUTECs: Primary human tubular epithelial cells, HK-2: Human kidney-2, HPTCs: Human primary renal proximal tubule cells, RPTECs: renal proximal tubular epithelial cells, hESCs: embryonic stem cells, HUVEC: human umbilical vein endothelial cells, NKi-2: human renal epithelial cells, MDCK: Madin-Darby canine kidney, MRC-5: human fibroblasts, MEK: mouse embryonic kidney, HK-2: human immortalized proximal tubule epithelial cells, ciPTECs: Conditionally immortalized proximal tubule epithelial cells, HRECs: Human renal epithelial cells, LLC-PK1: pig kidney epithelial cells, 3T3: fibroblasts, UB: ureteric bud,HEK-293: Human embryonic kidney cell line, CaKi-1: human renal cancer cells, mIMCD: mouse inner medullary collecting duct, GMECs: glomerular microvascular endothelial cells, HKC-8: human proximal tubular epithelial cells, WS-1: human dermal fibroblasts, hBMMSCs: human bone marrow-derived mesenchymal stem cells, GMECs: glomerular microvascular endothelial cells, iRECs: induced renal tubular epithelial cells.

### Extracellular matrix sources for *in vitro* models

*In vivo*, stromal cells (fibroblasts) produce ECM, and renal epithelial cells contribute themselves to BM synthesis, which can be exploited in *in vitro* models (Satyam et al., 2020). Simple ECM proteins like laminins are commercially available as recombinant proteins with proven utility for *in vitro* kidney models (Karamessinis et al., 2002; Chung et al., 2008; Zhang et al., 2009; Sebinger et al., 2013; Homan et al., 2019; Adelfio et al., 2020). However, most *in vitro* research uses ECM extracts from animal tissues because it is difficult to make complex macromolecular GAGs and large proteoglycans (Petkau-Milroy and Brunsveld, 2013; Aisenbrey and Murphy, 2020; Xing et al., 2020). Stroma-rich tissues like bone or cartilage can yield large quantities of pure ECM components. Commercial sources for human and animal collagen in various isoforms, hyaluronic acid, and fibronectin are available.

The biochemical complexity present in the original tissue is required to induce or maintain a specific phenotype. Here it is best to use complex, unfractionated tissue extracts containing a complex mix of glycosaminoglycans and other signaling molecules. Several commercial products, the best known being Matrigel (Kleinman and Martin, 2005; Passaniti et al., 2021), are readily available in different formulations (e.g., reduced growth factors). There are many examples of *in vitro* renal models employing such extracts (Zhang et al., 2009; Lam et al., 2014; Takasato et al., 2015; Figliuzzi et al., 2017; King et al., 2017; Hiraki et al., 2018; Howden et al., 2019; Otero et al., 2020) (Supplementary Table S1). Matrigel complexity (contains laminin, collagen IV, entactin, heparan sulfate proteoglycan and bound growth factors) yields better results than gelatin, collagen I, poly-L-lysine, and laminin alone (Hughes et al., 2010; Gao et al., 2011; Passaniti et al., 2021).

Matrigel-like products are expensive, batch-variable, ethically questionable (made from tumors grown in animals) and cannot be employed in human cell-therapy downstream applications. To solve recent availability and ethics-related issues, JellaGel, made from jellyfish Collagen 0 isolates, has recently become available. However, this formulation does not fully mimic the kidney ECM’s specific proteomic signature. ECM extracts from decellularized human kidneys can capture this specificity (Figliuzzi et al., 2017; Hiraki et al., 2018).

In recent years, artificial ECMs have been designed to replace natural ECM for renal epithelium scaffolding to reduce batch variability and degradation. This alternative allows more control over biochemical and mechanical properties and functionalization with instructive biomolecular tags to enhance cell attachment, proliferation, and differentiation (Aisenbrey and Murphy, 2020). Synthetic ECMs can be formed as hydrogels (Minuth et al., 2004; Chung et al., 2008; Astashkina et al., 2012) or hollow fibers (Dankers et al., 2011; Jansen et al., 2015; Shen et al., 2015; Chevtchik et al., 2016; Jansen et al., 2019; Xie et al., 2020; Myram et al., 2021) (Supplementary Table S1). The goal is to obtain a material whose composition can be tailored to control physiochemical matrix properties such as elasticity (Love et al., 2019), density, and stiffness, while ensuring low degradation under specific conditions (Petkau-Milroy and Brunsveld, 2013; Cruz-Acuña et al., 2019).

## Conventional models of kidney function and disease

### Two-dimensional renal cell culture on extracellular matrix-Coated surfaces

**FIGURE 1:**
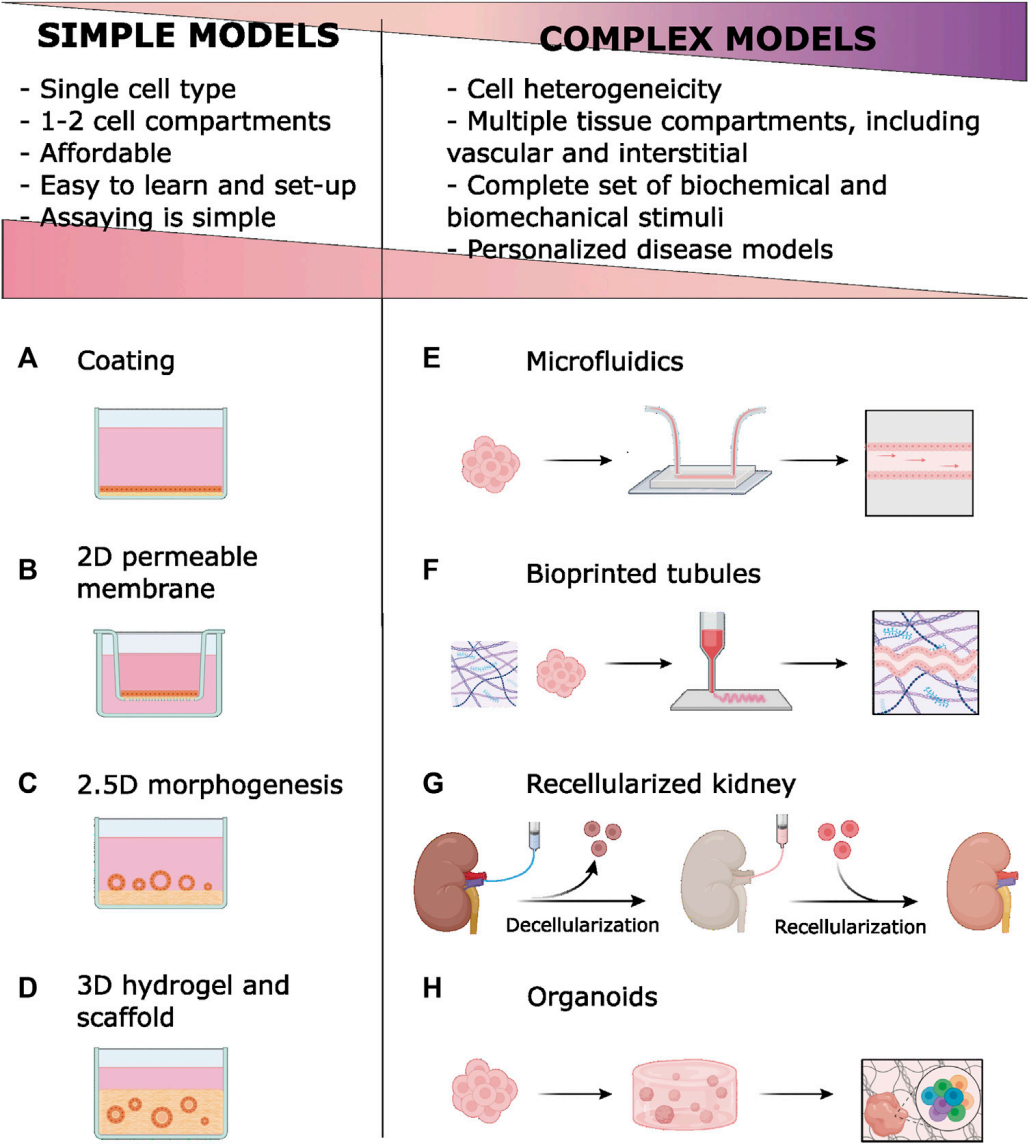
Simple models *in vitro* of renal epithelia employ ECM components of basal membrane (BM) and extracellular matrix (ECM) Coatings are simply ECM materials, usually collagen, adsorbed to the plastic **(A)** or permeable membrane **(B)**. Permeable membranes and scaffold-based models improve epithelial polarity by offering two fluid compartments. Tubulogenesis is stimulated by growing renal epithelial cells onto **(C)** or within **(D)** hydrogels. New technologies [microfluidics **(E)**, bioprinting **(F)**, ECM decellularization **(G)** and organoids development **(H)**] enable complex models that introduce cell heterogeneity, vascular and interstitial compartments, and biomechanical stimuli. Compared to simpler, conventional models, these models have disadvantages related to their complexity. However, complex models allow for more faithful modeling of kidney function and disease. A licensed version of BioRender was used to prepare this figure.

In the simplest culture configuration, renal cells grew directly on plastic surfaces as two-dimensional (2D) epithelial monolayers (Figure 1A). Adsorbing (coating) ECM components on plastic surfaces enhances renal cell adhesion, proliferation, and differentiation (Narayanan et al., 2013; van Gaal et al., 2021). Matrigel’s complex set of biochemical signals is used when cell differentiation is the goal (Narayanan et al., 2013). ECM coatings are also used to functionalize synthetic scaffolds (Chung et al., 2008; Ni et al., 2011). Relevant examples of 2D *in vitro* kidney models are provided in Table 1. However, lack of complex cell interactions can lead to undesired effects, such as epithelial-to-mesenchymal transition (EMT) (Forino et al., 2006).

Epithelial 2D monolayers are frequently grown on permeable supports, like the Transwell system, to promote cell polarization (Gao et al., 2011; Ni et al., 2011; Shamir and Ewald, 2014; Schophuizen et al., 2015; Hulshof et al., 2018) (Figure 1B). Porous membranes can be coated with ECM (Ni et al., 2011; Shamir and Ewald, 2014) or used as scaffolds for thin hydrogels to improve mechanical properties (Shamir and Ewald, 2014) (Table 1). This configuration also facilitates co-culture with other kidney-relevant components (King et al., 2017).

When grown on top of hydrogels, kidney primary cells (Zhang et al., 2011) and most renal cell lines form tubular structures (tubulogenesis; termed 2.5D architecture; Figure 1C). Formation of tubules requires adding Matrigel to the hydrogel and/or to the medium, stressing the need for specific ECM chemical signals (Shamir and Ewald, 2014; Bosch-Fortea et al., 2019). Tubulogenesis studies on the MDCK cell line have been instrumental for understanding molecular the processes involved in epithelial differentiation and polarization (Bosch-Fortea et al., 2019). Hagelaars et al. have recently used this model to show cellular differences in how matrix stiffness affects integrin-mediated cell-ECM attachment and cell polarization (Hagelaars et al., 2022). Better differentiation can happen when the tubule is fully embedded in the ECM (3D architecture, discussed below), however, direct exposure to culture medium in 2.5D structures facilitates imaging and biochemical treatments and assays.

### Three-dimensional renal cell culture in hydrogel and scaffolds

Primary or continuous renal cell lines grown within collagen I or Matrigel hydrogels spontaneously form tubule-like structures (Taub et al., 1990; Sakurai et al., 1997; Zegers et al., 2003; Joraku et al., 2009; Schlüter and Margolis, 2009) (Figure 1D). Dissociated cells are mixed with biocompatible hydrogels in liquid form and allowed to polymerize (Miya et al., 2011; DesRochers et al., 2013). Alternatively, cells can be sandwiched between two ECM layers (Montesano et al., 1991a; Montesano et al., 1991b; Kher et al., 2011; Secker et al., 2018; Dixon et al., 2020) In the presence of appropriate factors, cells form hollow cysts (Zegers et al., 2003; Schlüter and Margolis, 2009) and continue to differentiate by elongation (Joraku et al., 2009) and tubule branching. Different nephron segments, including the glomerulus, have been modeled in 3D hydrogels (Joraku et al., 2009; Lü et al., 2012; Hirashima et al., 2017).

3D-culture allows co-culturing of different renal cell lineages, introducing complexity in kidney models (Montesano et al., 1991a; Montesano et al., 1991b; Secker et al., 2018). Wang et al. showed that using a sandwich 3D co-culture model is possible to recreate *in vitro* the tubule-interstitial-vascular unit, with more tubular cell polarity and enhanced functional gene expression (Wang et al., 2020). Gelatin-fibrin hydrogels outperformed conventional Transwell co-culture in modeling the proximal tubule-vascular unit *in vitro* (Lin et al., 2019). Tubulointerstitial fibrosis, a common feature in chronic kidney disease, has been modeled in 3D co-cultures (Moll et al., 2013).

Collagen I and Matrigel-like ECM extracts are often used, alone, mixed, or combined with other ECM components like collagen IV, fibronectin, or laminins (Weber et al., 2017). Functionality depends on the 3D hydrogel’s composition, protein concentration, and stiffness (Shamir and Ewald, 2014; Hirashima et al., 2017; Hiraki et al., 2018). Hyaluronic acid is an interesting alternative to tissue extracts because it provides good mechanical and biochemical stimuli. 3D organoids containing proximal tubule structures were generated from mouse kidney explants by prolonged (6 weeks) culture in hyaluronic acid hydrogels resembling *in vivo* environment (Astashkina et al., 2012). This model has been successfully employed in the preclinical evaluation of nanoparticle nephrotoxicity (Astashkina et al., 2014). Hyaluronic acid modulated ureteric bud branching and promoted mesenchymal-to-epithelial transition (Rosines et al., 2007). The polarity of tubular structures obtained by growing human renal cells (HK-2, ciPTEC, and primary proximal tubule cells) in glycosaminoglycan-based hydrogels was found to depend on sulphated GAGs (Weber et al., 2017). Matrix stiffness strongly affects tubulogenesis in MDCK cells (Hirashima et al., 2017). Such studies (Table 1) exemplify how morphogenesis and function can be modulated by adjusting hydrogel degradability, growth factor signaling, and mechanics.

ECM-derived hydrogels have low resistance to mechanical stress, partly a consequence of active cell remodeling, limiting their use. A potential solution is to exploit the mechanical properties of natural (silk, alginate) or synthetic (PEG, PCL, PLA) polymers to complement natural ECM components (Chung et al., 2008; Enemchukwu et al., 2016; Gupta et al., 2019) (Supplementary Table S2). Plastic materials are easily deposited in 2D or 3D structures by electrospinning or printing techniques (Dankers et al., 2011; Sobreiro-Almeida et al., 2020; van Genderen et al., 2021). Murine PKD1 knockout renal tubular cells seeded in silk-scaffolds filled with Matrigel and collagen hydrogels has been shown to reproduce morphological and functional abnormalities present in Autosomal Dominant Polycystic Kidney disease (ADPKD) (Subramanian et al., 2010; Subramanian et al., 2012).

Recently, 3D models have gained momentum with microfabrication techniques. The organ-on-a-chip technology aims to mimic *in vivo* tissue architecture by providing independent but connected compartments (Rayner et al., 2018) (Figure 1E). Models based on microfabricated devices recapitulate intercellular and cell-ECM interactions at the microscale. Microfluidics integration adds flow-mediated shear stress, a critical mechanical stimulus for the renal tubule (Jang et al., 2013). Mimetas Organoplate allows for a high throughput culture and analysis of 3D tissue units under fluidic stimulus (Schutgens et al., 2019) and it has proven useful in modeling nephrotoxicity (Vormann et al., 2021) and acute kidney injury (Vormann et al., 2022). Commercial organ-on-chip solutions are listed in Supplementary Table S2.

3D models of tubulogenesis have been instrumental in defining chemical and mechanical stimuli involved in ECM-cell interactions and their role in kidney development and function. Lumen access, high-resolution imaging, biochemical studies, and manipulation for functional or nephrotoxicity assays are, however, limited in their architecture.

## New strategies in *in vitro* modeling of kidney function and disease

### Bioprinted scaffolds

Bioprinting has recently emerged as a tool for building complex tissue structures. Biocompatible polymers (bioinks) are layered to create 3D structures (Figure 1F). Cells are seeded on these 3D scaffolds or directly mixed in the bioink. Bioprinting offers unprecedented flexibility and versatility to recreate *in vivo* environments at the microscale through stereotaxic control of bioink deposition (Fransen et al., 2021).

Bioinks are chosen for their rheological properties and printing device compatibility. As technology evolves, the use of bioinks derived from native tissue like collagen and decellularized ECM is favored (Garreta et al., 2017; Ali et al., 2019; Dzobo et al., 2019; Sobreiro-Almeida et al., 2021). This enhances kidney-specific gene expression by providing tissue-specific biochemical cues (Singh et al., 2020). However, because ECM bioinks exhibit poor mechanical stability, they are often combined with other polymers like methacrylate (Ali et al., 2019).

Using bioprinting, perfused renal tubules have been successfully made. Lewis’s group used fugitive ink to cast tubular conduits within hydrogels, which they populated with proximal tubule cells or endothelial cells and perfused in a closed circuit for days. These tubular-vascular units expressed differentiated phenotypes, and their response to pathogenic insults mimicked those observed in native human tissues (Homan et al., 2016; Lin et al., 2019; Aceves et al., 2022). Tröndle et al. recently reported a modified 3D sandwich model in which renal cells were bioprinted as clusters at a controlled topography on a collagen and Matrigel substrate gel. Cell clusters formed lumen-containing spheroids, which coalesced into tubular structures that could be connected to fluidic systems (Tröndle et al., 2021). Both strategies use fibrin polymers to improve hydrogel biomechanics. A third strategy used a proprietary bioprinting technology (the Organovo 3D printing platform) to sequentially print epithelial tubule, fibroblast-containing ECM, and endothelial vessels (King et al., 2017). A glomerular functional unit was successfully recreated by printing hollow tubules from a functional hybrid bioink (alginate plus decellularized ECM) (Singh et al., 2020).

Bioprinting’s flexibility and automatization capabilities make it a promising method for *in vitro* modeling of the kidney’s basic functional unit. An immediate challenge is to make it affordable for the general laboratory.

### Decellularized kidney as a tissue-specific scaffold

Regenerative medicine has long sought to fabricate a functional kidney using a donor’s decellularized ECM scaffold repopulated with host cells to mitigate the shortage of organs available for transplant (Sullivan et al., 2012). The technique involves perfusing whole kidneys with detergent solutions to remove cells and preserve ECM microscopic architecture and tissue-specific ECM components like collagens and laminins, as well as basement membranes (Song et al., 2013) (Figure 1G). Conservation of signaling molecules, namely glycosaminoglycans, requires proper detergent composition and perfusion rates and timing (Caralt et al., 2015; Poornejad et al., 2016; He et al., 2017; Kajbafzadeh et al., 2019; Zhou et al., 2020; Shahraki et al., 2022). Decellularization can be successfully applied to stored frozen tissues (Chani et al., 2017). Decellularized scaffolds have been successfully repopulated with pluripotent, progenitor, epithelial, or endothelial cells. When implanted in animal models, this bioengineered tissue integrates with host structures and shows some kidney functions (Bonandrini et al., 2014; Caralt et al., 2015; Figliuzzi et al., 2017; Ciampi et al., 2019; Han et al., 2019; Zhang et al., 2019) or helps to revert EMT and fibrosis (Hu et al., 2020).

Regenerating a fully functional organ from a decellularized scaffold is a formidable challenge because of the kidney’s high structural and functional complexity. Nevertheless, studies on kidney decellularization have provided valuable information on cell-ECM interactions, supporting GAGs’ critical role (Louzao-Martinez et al., 2019; Ullah et al., 2020). Moreover, decellularized scaffolds are useful for *in vitro* method development. Decellularized kidney sections serve as scaffolds for growing renal cells in nephrotoxicity models (Fedecostante et al., 2018). These scaffolds allow for the investigation of cell-ECM interactions in specific organ or tissue microdomains. For example, the fate of pluripotent or progenitor cells in a recellularized scaffold can be followed to learn about specific cell differentiation determinants (Du et al., 2016; Bombelli et al., 2018; Zhang et al., 2019; Bombelli et al., 2020; Ullah et al., 2020).

An acid hydrolysate of decellularized kidney scaffolds, termed dKECM, can be used as a source of tissue-specific ECM materials for surface coating and hydrogel fabrication (Hiraki et al., 2018; Zhou et al., 2020; Shen et al., 2021; Lee et al., 2022). Combining dKECM with other natural or synthetic compounds can enhance their rheological or biophysical properties (Lih et al., 2019; Sobreiro-Almeida et al., 2019; Sobreiro-Almeida et al., 2020; Geng et al., 2021; Ko et al., 2021; Sobreiro-Almeida et al., 2021). Accordingly, dKECM is becoming a favorite bioink in bioprinting applications (Ali et al., 2019; Han et al., 2019). The undesired effects observed when growing human glomerular endothelial cells within hydrogels made of porcine dKECM (Su et al., 2018) illustrates the remarkable specificity of biochemical signals delivered by ECM.

### Role of ECM in kidney organoids development

Two strategies are currently used to develop 3D renal structures from progenitor or pluripotent cells by exploiting kidney development programs. Tubuloids are generated from primary cells and kidney organoids from pluripotent stem cells. Both situations require ECM components. Tubuloid culture is a refined version of 3D culture in Matrigel hydrogels where specific biochemical factors are added to stimulate progenitor cell proliferation and differentiation (Schutgens et al., 2019; Wiraja et al., 2021). Human tubuloid culture allows for long-term propagation of donor-specific primary kidney epithelium without requiring immortalization or genetic modification. A recent study comparing the polarization of tubuloid-derived cells and MDCK cells in response to substrate stiffness demonstrated tubuloid-derived cells appear to have different requirements and use different polarization mechanisms (Hagelaars et al., 2022). Unlike tubuloids, immortalized, well-established cell lines have been selected to grow on plastic substrates. By skipping the phase of culture on a stiff substrate, tubuloids might retain more of their physiological responses to ECM. This makes them a simple and affordable alternative cell source for *in vitro* models.

In the organoid technique (Figure 1H), Matrigel (Xia et al., 2013; Kang and Han, 2014; Takasato et al., 2015; Takasato and Little, 2017; Howden et al., 2019; Low et al., 2019) or Geltrex (Lam et al., 2014; Morizane et al., 2015; Morizane and Bonventre, 2017) coatings or hydrogels are used in feeder-free culture of stem cells or at several differentiation steps. For instance, Taguchi et al. used a 50% Matrigel culture medium to stimulate branching morphogenesis in ureteric buds and to induce interactions with nephron progenitors (Taguchi and Nishinakamura, 2017). Freedman et al. induced epiblast spheroids differentiation by sandwiching hPSC between two layers of diluted Matrigel (Freedman et al., 2015). Under the appropriate concentration and timing of specific biochemical inducers, complex self-organized 3D structures develop.

Organoids contain kidney parenchyma and stroma components, and the synthesis of ECM has been observed (Lam et al., 2014; Takasato et al., 2015; Howden et al., 2019). Given the complexity and animal origin of Matrigel and similar products, there have been efforts to replace it with recombinant ECM proteins, such as laminins (Howden et al., 2019; Mae et al., 2020) or vitronectin (van den Berg et al., 2018), or synthetic products like Synthemax (Toyohara et al., 2015). Recently, Geunes et al. cultured kidney organoids in thiol-ene cross-linked alginate hydrogels and showed a reduction in the onset of aberrant ECM expression and off-target cell populations (Geuens et al., 2021). By engineering gel mechanics and dynamics, ECM deposition and organoid maturation could be tuned, highlighting the role of engineered matrices in stirring organoid commitment (Ruiter et al., 2022).

Incomplete maturation and lack of vascularization are unsolved issues in organoid development where a proper selection and use of ECM components could help. Garreta et al. demonstrated that ECM biophysical properties modulate hPSC proliferation and differentiation (Garreta et al., 2019). Soft hydrogels with stiffness in the physiological range better mimic the early stages of embryonic development. Vascular compartment expression improved when organoids were grown in hydrogels made from decellularized human kidney extracellular matrix (Kim et al., 2022). Bioprinting cellular bioinks allows for precise and reproducible manipulation of organoid size and more differentiated cells (Howden et al., 2019; Lawlor et al., 2021). Perfusion of organoids in microfluidic devices induces higher expression of vascular and podocyte compartments (Homan et al., 2016; Lee et al., 2021). High levels of structural and functional complexity in bioprinted, perfused organoids model more faithfully renal function and disease, as shown recently for APKD (Howden et al., 2021; Hiratsuka et al., 2022).

## Relevance of ECM-based *in vitro* models of renal disease for studies of renoprotection and kidney regeneration

Increasing rates of chronic kidney disease (CKD) represent a major burden for social and healthcare systems worldwide. Fighting underlying causes (diabetes, obesity, cardiovascular disease, etc.) is key. But it is equally important to prevent, slow down, or reverse CKD progression, which very often results from maladaptive responses to acute kidney injury.

Shortcomings of traditional preclinical models (animal experimentation and conventional cell culture) have fueled the development of sophisticated *in vitro* kidney models that take advantage of recent technological advances (Morizane et al., 2015). Only through such complex models is it possible, for instance, to recreate the delicate glomerular filtration barrier, allowing for the investigation of the varied glomerulopathies (Lü et al., 2012; Du et al., 2016; Xie et al., 2020). Sophisticated models, such as organoids, are already being used successfully to study genetic (e.g., ADPKD (Subramanian et al., 2010; Freedman et al., 2015; Dixon et al., 2020; Zhang et al., 2020; Howden et al., 2021; Myram et al., 2021; Hiratsuka et al., 2022)) or metabolic tubulopathies (Fabry’s disease (Kim et al., 2022)). Investigating the mechanisms of drug-related nephrotoxicity and discovering ways to prevent it is frequently the goal behind model design or validation (Astashkina et al., 2012; DesRochers et al., 2013; King et al., 2017; Fedecostante et al., 2018; Vormann et al., 2021; Tröndle et al., 2022). Research on common mechanisms underlying CKD progression, irrespective of its cause, such as epithelial-to-mesenchymal transition (Forino et al., 2006) or fibrosis (Moll et al., 2013; Hu et al., 2020; Li et al., 2022), requires the presence of all participants in such complex processes. Studying tubulogenesis or cell-repair mechanisms in complex *in vitro* models helps identification of signals needed for kidney regeneration (Miya et al., 2011). These processes, which involve multiple actors from distinct compartments, cannot be studied adequately with conventional *in vitro* models. Some bioengineering strategies are originally aimed at fabricating tissue-like structures for regenerative techniques, based on the concept of regenerating an entire organ from a decellularized scaffold (Song et al., 2013; Du et al., 2016; Figliuzzi et al., 2017; Ciampi et al., 2019) or by stimulating regeneration *in vivo via* cell or tissue implants (Lih et al., 2019; Ko et al., 2021; van den Berg et al., 2018; Garreta et al., 2019; Kim et al., 2022).

We have summarized the essential roles of ECM in *in vitro* kidney function and disease modeling. ECM provides the biochemical and mechanical stimuli required for promoting and maintaining cell differentiation. A proper 3D architecture also permits cell-cell interactions and facilitates the presence of all necessary compartments, including fluid convection, to faithfully mimic *in vivo* kidney function. More efforts are needed to make the technical skills required simpler and to lower the costs associated with using such models, which would increase their adoption in kidney translational research.
