# Supplementary material for: Role of extracellular matrix components and structure in new renal models in vitro
Source: Front Physiol. 2022 Dec 7;13:1048738. doi: 10.3389/fphys.2022.1048738 (PMC9767975; doi:10.3389/fphys.2022.1048738)
Supplement: Supplementary file 1 [file DataSheet1.pdf]

SUPPLEMENTAL MATERIALS

Role of extracellular matrix components and structure in new renal models *in vitro*

Alodia Lacueva-Aparicio[1,2], Rafael Soares Lindoso[3], Silvia M. Mihăilă[4], Ignacio Giménez[1,5,6]

Supplemental Figure 1

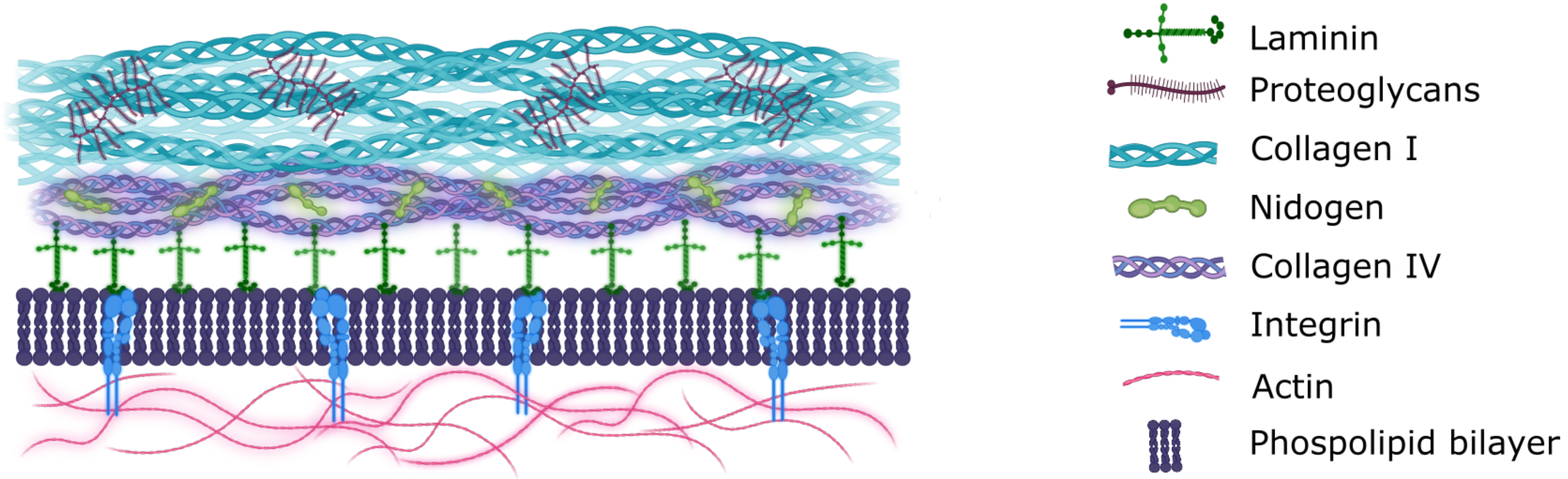

Figure S1. The extracellular matrix is mainly composed of proteoglycans, hyaluronic acid and collagen I. Proteoglycans are a core protein to which GAGs are attached, like hyaluronic acid. Proteoglycans have an important role in different biological functions, and they also interact with growth factors and take part in cell signaling. Collagen I is a fibril-forming collagen, and it is involved in the organization and the architecture of the specific tissue. Nidogen, collagen IV and laminins are part of the basal membrane. Laminins interweave with collagen IV network and nidogen acts as a connector or linking protein. Integrins are heterodimeric receptors that connect the ECM and the cytoskeleton. A licensed version of BioRender was used to prepare this figure.

Supplemental Tables

Supplementary Table 1. ECM components and their natural and synthetic surrogates

Component	Molecular nature	Application	References
Individual ECM/BM components			
Collagens			
• Collagen I	Isolated fraction, purified	2D Coating	(34,63)
		2.5 D	(11)
		3D Hydrogel	(23,68,74)
		Bioprinting	(105)
• Collagen IV	Isolated fraction, purified	2D Coating	(34,60)
• Jellyfish Collagen 0 (Jellagen)	Isolated fraction	3D scaffold	https://www.biotrend.com/en/brand/Jellagen
Laminins	Recombinant	Coating	(34,60)
Fibronectin	Isolated fraction, purified	Coating	(60)
GAGs			
• Hyaluronic Acid	Isolated fraction, purified	3D hydrogel	(85)
• Heparin	Isolated fraction, purified	3D hydrogel	(17)
Native ECM/BM extracts			
Matrigel	Unfractionated tissue Extract	2.5D	(11,81)
		Coating	(60)

		3D Hydrogel	(67)
Geltrex	Unfractionated tissue Extract	Coating	(139,141)
NovoGel		Bioprinting	(46)
Specific Renal ECM	Decellularized kidney	Recellularization	(43,114)
		ECM extracts	(88)
		Bioinks	(100,101)
Natural polymers			
Gelbrin		3D Scaffold	(32)
Silk		3D Scaffold	(86,90,91)
Synthetic polymers			
PEG	Monomer solution	Hydrogel	(30,87)
starPEG	Monomer solution	Hydrogel	(17)
EDC	Monomer solution	Hollow fiber hydrogel	(55)
microPES	Monomer solution	Membrane<br>HFM	(52,56)
PCLdi(u-UPy)	Monomer solution	Electrospun membrane	(51)
PES	Monomer solution	Membrane	(13,65)
PCL	Monomer solution	Electrospun polymer scaffold	(57)
Polyester fleece	Monomer solution	Scaffold	(50)
h- FIBER	Monomer solution	HFM	(54)

ECM: Extracellular Membrane, BM: Basal Membrane; GAGs: glycosaminoglycans
Matrigel: murine EHM tumor tissue, Gelbrin: Gelatin-Fibrin, PEG: polyethylene glycol, PEGDA: poly (ethylene glycol) diacrylate biofunctional polymer, EDC: 1-ethyl-3-(3- (dimethylamino)propyl) carbodiimide hydrochloride, HFM: hollow fiber membrane, PES: Polyethersulfone, PCL: polycaprolactone, h-FIBER: Extruded topographic hollow fiber

Supplementary Table 2. Commercial solutions for organ-on-a-chip technology

Company	Internet address	Kidney model*
4Dcell	https://www.4dcell.com/	No
AlveoliX	https://www.alveolix.com/	No
AxoSim	https://axosim.com/	No
BEOnChip	https://beonchip.com/	Yes
BioIVT	https://bioivt.com/	No
BiomimX	https://www.biomimx.com/	No
Cherry Biotech	https://www.cherrybiotech.com/	No
Elvesys	https://www.elveflow.com/	No
Emulate	https://emulatebio.com/	Yes
Ibidi	https://ibidi.com/	No
Insphero	https://insphero.com/	No
MesoBioTech	https://mesobiotech.com/	No
Mimetas	https://www.mimetas.com/en/home/	Yes
Nortis Bio	https://nortisbio.com/	Yes
TARA Biosystems	https://tarabiosystems.com/	No
TissUse	https://www.tissuse.com/en/	No

*Company specifically offers devices or models to model kidney tubule function.
